# Individual stiffness optimization of dorsal leaf spring ankle–foot orthoses in people with calf muscle weakness is superior to standard bodyweight-based recommendations

**DOI:** 10.1186/s12984-021-00890-8

**Published:** 2021-06-08

**Authors:** Niels F. J. Waterval, Merel-Anne Brehm, Jaap Harlaar, Frans Nollet

**Affiliations:** 1 Rehabilitation Medicine, Amsterdam Movement Sciences, Amsterdam UMC, University of Amsterdam, Meibergdreef 9, Amsterdam, The Netherlands; 2 Department of Biomechanical Engineering, Delft University of Technology, Delft, The Netherlands; 3 Department of Orthopaedics, Erasmus Medical Center, Rotterdam, The Netherlands

## Abstract

**Background:**

In people with calf muscle weakness, the stiffness of dorsal leaf spring ankle–foot orthoses (DLS-AFO) needs to be individualized to maximize its effect on walking. Orthotic suppliers may recommend a certain stiffness based on body weight and activity level. However, it is unknown whether these recommendations are sufficient to yield the optimal stiffness for the individual. Therefore, we assessed whether the stiffness following the supplier’s recommendation of the Carbon Ankle7 (CA7) dorsal leaf matched the experimentally optimized AFO stiffness.

**Methods:**

Thirty-four persons with calf muscle weakness were included and provided a new DLS-AFO of which the stiffness could be varied by changing the CA7® (Ottobock, Duderstadt, Germany) dorsal leaf. For five different stiffness levels, including the supplier recommended stiffness, gait biomechanics, walking energy cost and speed were assessed. Based on these measures, the individual experimentally optimal AFO stiffness was selected.

**Results:**

In only 8 of 34 (23%) participants, the supplier recommended stiffness matched the experimentally optimized AFO stiffness, the latter being on average 1.2 ± 1.3 Nm/degree more flexible. The DLS-AFO with an experimentally optimized stiffness resulted in a significantly lower walking energy cost (− 0.21 ± 0.26 J/kg/m, *p* < 0.001) and a higher speed (+ 0.02 m/s, *p* = 0.003). Additionally, a larger ankle range of motion (+ 1.3 ± 0.3 degrees, *p* < 0.001) and higher ankle power (+ 0.16 ± 0.04 W/kg, *p* < 0.001) were found with the experimentally optimized stiffness compared to the supplier recommended stiffness.

**Conclusions:**

In people with calf muscle weakness, current supplier’s recommendations for the CA7 stiffness level result in the provision of DLS-AFOs that are too stiff and only achieve 80% of the reduction in energy cost achieved with an individual optimized stiffness. It is recommended to experimentally optimize the CA7 stiffness in people with calf muscle weakness in order to maximize treatment outcomes.

*Trial registration* Nederlands Trial Register 5170. Registration date: May 7th 2015. http://www.trialregister.nl/trialreg/admin/rctview.asp?TC=5170.

**Supplementary Information:**

The online version contains supplementary material available at 10.1186/s12984-021-00890-8.

## Introduction

Persons with neuromuscular disorders like Charcot–Marie–Tooth disease and poliomyelitis often exhibit weakness of their calf muscles. Calf muscle weakness changes the gait pattern, and typically leads to excessive ankle dorsiflexion, persistent knee flexion and reduced ankle push-off power during stance [1, 2]. These gait deviations lower walking speed and elevate walking energy cost by − 30% and + 60%, respectively [1, 3].

To improve walking in persons with calf muscle weakness, dorsal leaf spring ankle–foot orthoses (DLS-AFOs) can be provided with the aim to restrict the ankle dorsiflexion angle by providing an external plantar flexion moment. This external moment is proportional to the bending and stiffness of the leaf spring [3–5]. If the ankle angle is successfully restricted, the ground reaction force can move further forward over the foot and in front of the ankle and knee [3, 6]. This reduces quadriceps activation, and, subsequently, walking energy cost [7, 8]. Additionally, DLS-AFOs can support the ankle power by storing energy when moving into dorsiflexion during the stance phase and releasing this energy during push-off, which also reduces walking energy cost [9].

The effects of DLS-AFOs on restricting the ankle dorsiflexion angle and supporting ankle power depends largely on the AFO’s ankle stiffness [5, 6, 10]. A higher AFO ankle stiffness restricts the ankle dorsiflexion angle more, though at the expense of the ankle power generating capacity, while a lower AFO ankle stiffness can enhance ankle power but reduces ankle dorsiflexion less effectively. Optimizing the trade-off between normalizing the ankle angle and preserving ankle power has been shown to maximize the reduction in energy cost [5, 6, 11]. Considering this trade-off is patient-dependent [5, 6, 11], it is necessary to individualize the DLS-AFO stiffness [8].

Previously, we demonstrated that individualization of the DLS-AFO stiffness in persons with calf muscle weakness resulted not only in a lower walking energy cost, but also in better treatment outcomes in terms of perceived fatigue and walking satisfaction, compared to AFOs provided in usual care [8]. In this particular study, the Carbon Ankle7® (CA7) leaf spring was optimized using objective experiments, despite the existence of a classification matrix based on the user’s body weight and activity level to individualize the stiffness of the CA7 leaf spring [12]. Individual optimization was motivated by the idea that besides body weight and activity level, other factors such as severity of weakness and walking speed likely influence the optimal stiffness [4, 5, 10]. The aim of this study was to assess whether the stiffness following the supplier recommendation matches the effects of an experimental selected optimal AFO stiffness for walking on level ground. Secondly, we want to study how differences in ankle power relate to walking energy cost, speed and hip power. We hypothesize that the supplier’s classification matrix will not result in the provision of the same AFO stiffness, and hence, result in less reduction in walking energy cost compared to an experimentally optimized AFO stiffness.

## Methods

For this study we used data from the PROOF-AFO trial, which was an observational study on the effect of optimizing the AFO ankle stiffness in people with calf muscle weakness [13]. The protocol of the PROOF-AFO trial was approved by the medical ethics committee of the Academic Medical Center (AMC) in Amsterdam, The Netherlands, and registered at the Dutch trial register with number NTR5170.

The main inclusion criterion for the PROOF-AFO trial was the presence of non-spastic calf muscle weakness (unilateral or bilateral) due to a neuromuscular disease or nerve damage. Other inclusion criteria were: aged 18 years or older, using an AFO or orthopedic shoes in daily life; able to walk for at least 6 min; and weight below 120 kg as this was the maximum for the intervention AFO according to the suppliers recommendation. Exclusion criteria were knee extensor weakness, which required use of a knee–ankle–foot orthosis and not being able to reach more than 0 degrees of ankle dorsiflexion (pes equinus) during weight bearing.

### Intervention

**Fig. 1:**
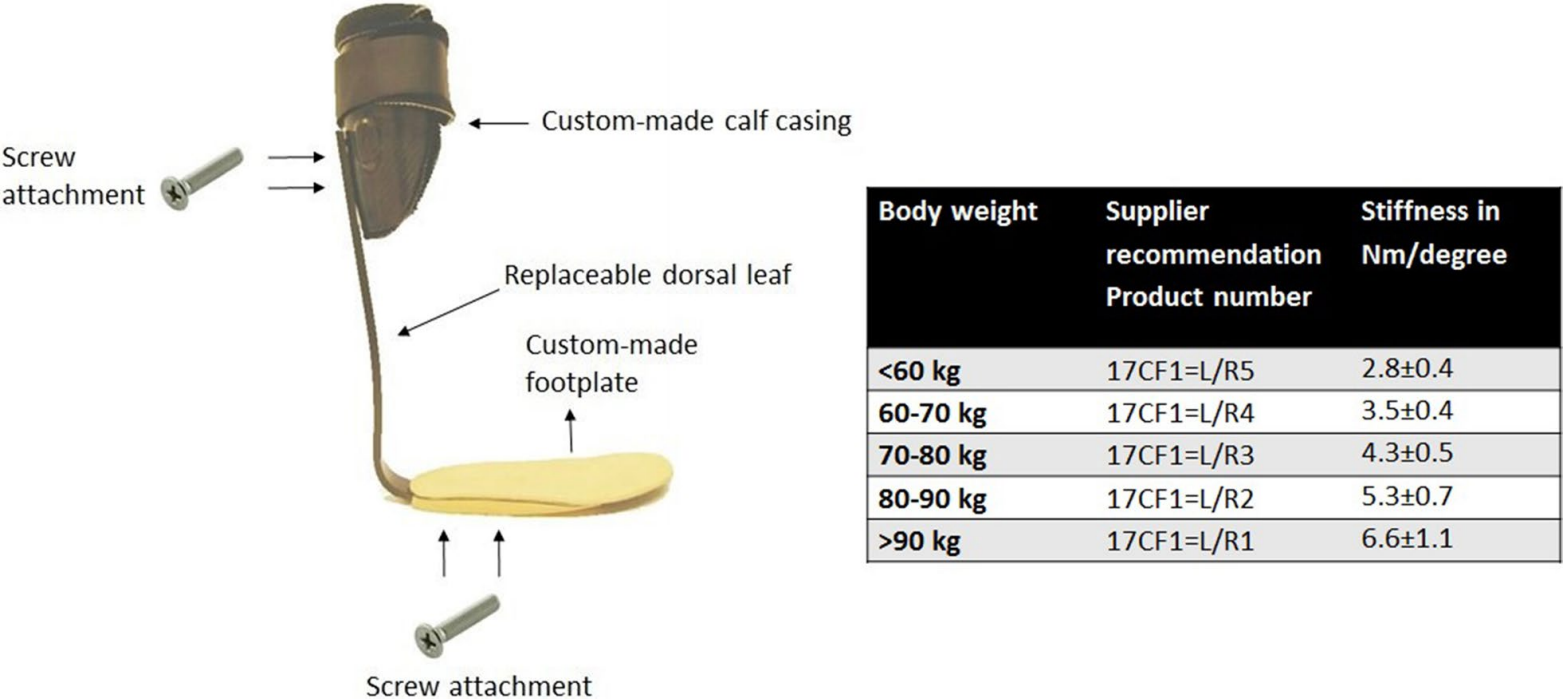
The intervention AFO. Table: subscription scheme from the supplier. The stiffness could be varied by changing the dorsal leaf

Participants were provided a new DLS-AFO (Fig. 1), for which we used the CA7® (Ottobock, Duderstadt, Germany). The CA7 leaf spring was attached to a custom-made calf casing and footplate using a total of four screws. This allowed us to alter the AFO stiffness by manually changing the CA7 leafs. For each participant, five different CA7 leafs, which all had a width of 30 mm (K1 [Ottobock classification: 17CF1 = L/R5, stiffness: 2.2 Nm/degree] to K5 [Ottobock classification: 17CF1 = L/R1, stiffness: 6.6 Nm/degree]) were tested [14]. Stiffness of the AFOs was measured with the Bi-articular Reciprocal Universal Compliance Estimator (BRUCE), which is a device specifically designed to reliably measure AFO characteristics [14]. To measure the stiffness, the AFO was strapped to the BRUCE dummy leg and manually moved towards dorsiflexion three times. During the movement, BRUCE recorded the ankle angle and the exerted moment. The AFO stiffness was calculated by dividing the ankle angle by the exerted moment.

### Measurements

#### Walking energy cost and comfortable speed

Walking energy cost and speed while walking without AFO and with the five AFO stiffness levels were measured during a 6-min walk test on a 35-m oval track. During the test, oxygen consumption (VO_2_) and carbon dioxide production (VCO_2_) were simultaneously measured (Cosmed K4B^2^, Rome, Italy). If needed, participants were allowed to use an assistive device, e.g. crutch or cane. To avoid interference with the test, participants were instructed to withhold the intake of food and sugar holding beverages in the two hours before the test.

#### Gait biomechanics

A 3D-gait analysis at comfortable speed while walking with the five AFO stiffness levels was performed to assess gait biomechanics. Markers were placed according to the PlugInGait model. Marker trajectories were recorded with a 8-camera 100 Hz Vicon MX 1.3 system (VICON, Oxford, UK), while ground reaction forces were measured using four force plates (1000 Hz, OR6-7, AMTI, Watertown, USA). Measurements for each stiffness condition were repeated until three valid trials were recorded (i.e. foot placed completely within a force plate and markers visible from heel strike on the force plate to ipsilateral heel strike).

#### Manual muscle strength

Manual muscle strength of the plantar flexors, dorsiflexors and knee extensors was assessed by a trained physician, and scored according to the Medical Research Council (MRC) scale [15].

### Data analysis

#### Walking energy cost and comfortable speed

To calculate walking energy cost, a steady-state period of at least 60-s during which VO_2_, VCO_2_ and speed were constant was visually determined for the last three minutes of the test. Walking energy cost was calculated by dividing the mean energy consumption (in J/kg/s, calculated according to Garby and Astrup [16]) by the average walking speed during the steady-state time-frame. The average walking speed during the steady-state period was considered the comfortable walking speed.

#### Gait biomechanics

The timing of foot-strike and toe-off were determined using force-plate data. Data were processed within VICON Nexus (VICON, Oxford, UK). Ankle angle, moment and power were time normalized (0–100% of the gait cycles) and averaged across trials using Matlab (The Mathworks, Natick, USA). Additionally, the maximum ankle angle, moment and power, and maximal positive hip power during early stance and pre-swing for each gait cycle were calculated. We selected the ankle parameters as they are clinically meaningful affected by AFO stiffness [5], while the hip power outcomes can provide insight into compensatory mechanisms.

### Selection of AFO stiffness

#### Experimentally selected optimal stiffness

The optimal CA7 AFO stiffness was individually selected based on a pre-defined optimization procedure. The selection was primarily based on walking energy cost as optimization metric and, secondarily, on walking speed and a clinical appraisal of the gait pattern by three independent assessors who were unaware of the supplier recommendations. In case the assessors selected different optimal AFO stiffness levels, the optimal stiffness was selected by consensus. An extensive description of the optimization procedure has been published elsewhere [13].

#### Supplier recommended stiffness

In clinical practice, the CA7 stiffness is determined on the basis of user’s body weight and activity level, according to a classification matrix as provided by the supplier, see Fig. 1 (Ottobock, Duderstadt, Germany). For each 10 kg body weight, a higher stiffness level is recommended. In addition, if patients are highly active it is advised to provide one stiffness level higher [12]. In this study, all participants were normally active.

### Data analysis

We analysed the agreement between the clinically selected AFO stiffness following the supplier classification matrix and the experimentally selected optimal AFO stiffness s with Cohen’s Kappa. Differences in energy cost and comfortable speed between walking with no AFO, supplier recommended AFO stiffness and the experimentally selected optimal AFO stiffness were tested with paired t-tests. Differences in effect-size between unilateral and bilateral affected patients were tested with independent t-tests.

Additionally, we divided participants in a group of high, medium and low responders to the experimental optimization based on their energy cost, in order to control whether a difference in stiffness explains the effects. In high responders, energy cost reduced by more than 10%, in medium responders energy cost reduced between 5 and 10%, while in low responders energy cost reduced less than 5%. Individual effects are presented in the Additional file 1: Table.

Differences between the supplier recommended AFO stiffness and experimental selected optimal AFO stiffness on ankle angle, moment and power were tested with a multilevel linear mixed model to account for the presence of unilateral and bilateral affected patients. The mixed model consisted of three levels; participant (third level), leg (second level), and condition (first level). To model differences between participants while walking with the supplier recommended AFO, a random intercept was incorporated, and to model differences in effect of the optimization a random slope was added. The analysis was performed in MLwiN 2.34 (Institute of Education, University of London, London, UK). For the ankle moment and power, participants walking with an assistive device during the gait analysis were excluded because no valid ground reaction forces could be measured.

To explain the difference in walking energy cost and speed between the supplier recommended and experimental optimized AFO stiffness, the relation between the difference in ankle power and difference in walking energy cost and speed between conditions was assessed with Pearson’s correlation. This was done for unilateral and bilateral affected subjects combined and separately. For bilateral affected subjects, the average difference in ankle power for the two legs was used. Participants for whom the orthotic-supplier recommended AFO stiffness and the experimental selected AFO stiffness were the same, were excluded from this particular analysis.

## Results

### Participants

Of the 37 participants included in the PROOF-AFO trial, three participants were left out from the analysis as the stiffness for their right and left leg were optimized separately. Sociodemographic and clinical characteristics of the remaining 34 participants are presented in Table 1. Twenty-two participants were bilaterally affected and used an AFO on both legs, of which two used a cane as assistive device.

**Table 1:** Sociodemographic and clinical characteristics of participants (n = 34)

*Sociodemographic characteristics*	
Age in years	56.9 ± 15.5
Gender male/female	20/14
Height in cm	178 ± 10
Weight in kg	85.6 ± 16.2
*Clinical characteristics*	
Diagnosis	Charcot–Marie–Tooth (n = 15)
	Poliomyelitis (n = 7)
	Nerve injury (n = 9)
	Myotonic dystrophy (n = 2)
	Myoshi distal myopathy (n = 1)
Unilateral/bilateral affected	12/22
MRC score legs with AFO/legs without AFO	
Plantar flexors	3 [2–4] / 5 [5]
Dorsiflexors	2 [1–4] / 5 [5]
Knee extensors	5 [5–5] / 5 [5]

*AFO* ankle–foot orthosis, *cm*  centimeter, *kg* kilogram, *MRC* Medical Research Council

#### Supplier recommended versus experimentally optimized AFO stiffness

The stiffness of the supplier recommended and experimental optimized AFO stiffness corresponded in 8 (23.5%) of 34 participants and differed in the remaining 26 participants (Table 2, kappa = 0.091, *p* = 0.187).

**Table 2:**
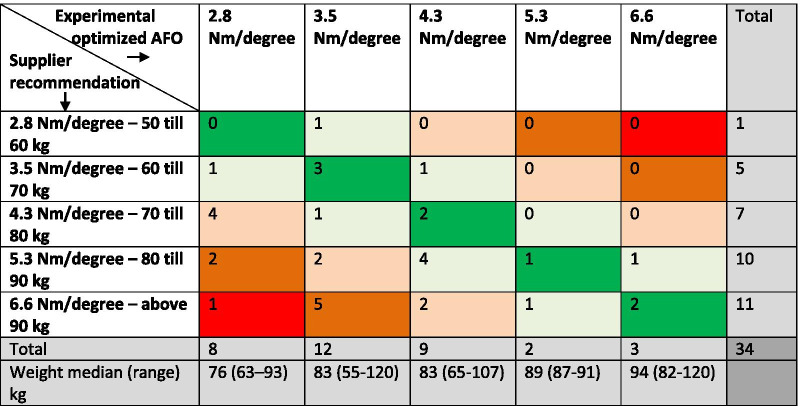
Difference in stiffness level between supplier recommended AFO and experimental optimized AFO

*AFO* ankle–foot orthosis, *kg* kilogram, green indicates that the experimental optimized and recommendation matched, while warmer (more red) colors indicate a larger difference in stiffness between the experimental optimized and recommended stiffness

In 20 (59%) of 34 participants the experimental optimized AFO stiffness was between 2 and 4 Nm/degree, in 9 (26%) participants it was 4.3 Nm/degree, while in only five participants it was above 5 Nm/degree (Table 2). These five participants all had a body weight above 80 kg and a walking speed below 1 m/s without AFO. On average, the supplier recommended AFO was 1.2 ± 1.3 Nm/degree higher compared to the experimentally optimized AFO stiffness (*p* < 0.001). This difference in stiffness was not significantly different between unilateral (1.1 ± 1.7) and bilateral (1.5 ± 1.2) affected participants (*p* = 0.457).

### Walking energy cost and speed

**Fig. 2:**
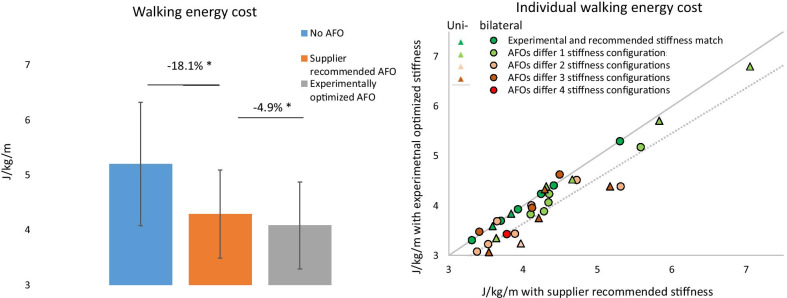
Effect of the supplier recommended and optimized AFO stiffness on walking energy cost. In the left panel, the average value for walking without AFO, with the supplier recommended AFO stiffness and experimental optimized AFO stiffness are presented. In the right panel, the individual differences between the recommended and optimized AFO stiffness are shown. The circles indicate people with bilateral calf muscle weakness, while the triangles indicates people with unilateral calf muscle weakness. The grey solid line represents no difference between the recommended and optimized AFO. The dotted line represents a 9% reduction (smallest detectable difference) with the optimized AFO stiffness compared to the recommended AFO stiffness. *AFO*  ankle–foot orthosis. * Dnotes a significant difference, *p* < 0.001

The experimental optimized AFO stiffness significantly reduced walking energy cost by an additional 4.9% compared to the supplier recommended stiffness (− 0.21 ± 0.26 J/kg/m supplier: 4.29 ± 0.79 J/kg/m vs experimental: 4.08 ± 0.78 J/kg/m, *p* < 0.001). This means that compared to walking without AFO the supplier recommended stiffness only achieves 82% of its potential effect on energy cost (without AFO: 5.24 ± 1.13 J/kg/m, supplier: to 4.29 ± 0.79 J/kg/m (*p* < 0.001), experimental: 4.08 ± 0.78 J/kg/m, *p* < 0.001) (Fig. 2). When excluding the participants for which the supplier recommended and experimental optimized AFO stiffness were matched, the effect increased to 6.2% (− 0.28 ± 0.27 J/kg/m, supplier: 4.37 ± 0.84 J/kg/m vs experimental: 4.10 ± 0.84 J/kg/m, *p* < 0.001). The improvement was not significantly different between unilateral (5.9%) and bilateral (4.2%) affected patients (*p* = 0.357).

Regarding walking speed, the supplier recommended AFO stiffness significantly increased speed by 0.21 ± 0.18 m/s (24.1%) compared to no AFO (from 0.87 ± 0.21 to 1.08 ± 0.18 m/s, *p* < 0.001). The experimental optimized AFO stiffness increased walking speed further to 1.10 ± 0.18 (+ 2.5%), which was significantly higher compared to the supplier recommended stiffness (*p* = 0.003). No difference in effect was found when excluding participants with the same recommended as experimentally optimized AFO or between unilateral (+ 3.6%) and bilateral (+ 1.8%) affected patients (*p* = 0.107).

At the individual patient level, six (18%) participants were high-responders, in whom energy cost reduced by 0.64 ± 0.2 J/kg/m while speed increased by 0.06 ± 0.05 m/s. In these cases, the experimentally optimized stiffness was 2.5 ± 0.5 Nm/degree lower compared to the supplier recommendation. Eight (23%) participants were medium-responders. In this group, energy cost reduced by 0.32 ± 0.05 J/kg/m and speed increased by 0.04 ± 0.05 m/s, while the experimentally optimized stiffness was 1.6 ± 1.0 N/m degree more flexible. Twelve (35%) participants were low-responders. Their energy cost reduced by 0.06 ± 0.12 J/kg/m and speed increased by + 0.00 ± 0.04 m/s and stiffness was 1.4 ± 1.7 Nm/degree lower compared to the supplier recommended stiffness.

### Gait biomechanics

No significant differences between the supplier recommended and experimental optimized AFO stiffness were found for maximal ankle dorsiflexion angle (*p* = 0.146) or maximal ankle moment (*p* = 0.716) during terminal stance. Ankle range of motion (*p* < 0.001) and ankle power (*p* < 0.001) were both significantly higher for the experimental optimized AFO stiffness compared to the supplier recommended AFO stiffness (see Table 3). No effects on compensatory maximal hip power during early stance and pre-swing were found (*p* > 0.248).

**Table 3:** Difference between supplier recommended AFO stiffness and experimental optimized AFO stiffness on ankle kinematics and kinetics

	Supplier recommended stiffness Β intercept Mean + S.E	Difference with experimental optimized stiffness Mean + S.E	Significance, *p*
Model = β + β1*condition			
Maximal dorsiflexion ankle angle	15.2 (0.8)	+ 0.8 (0.6)	0.146
Ankle range of motion in degree	15.0 (0.7)	+ 1.3 (0.3)	< 0.001
Maximal ankle moment	1.04 (0.04)	0.00 (0.01)	0.716
Maximal ankle power	1.32 (0.09)	+ 0.16 (0.04)	< 0.001
Maximal hip power early stance	1.03 (0.08)	+ 0.05 (0.10)	0.248
Maximal hip power pre-swing	1.09 (0.07)	+ 0.08 (0.12)	0.320

### Relation between change in ankle power and change in walking energy cost

**Fig. 3:**
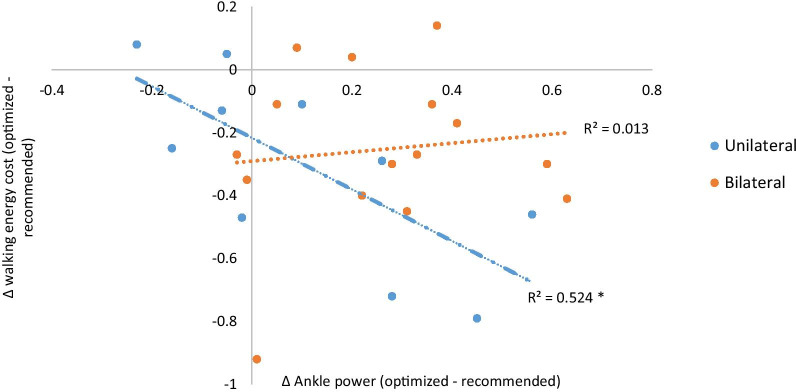
Relation between difference in ankle power and difference in walking energy cost between supplier recommended and experimentally optimized AFO stiffness for unilateral and bilateral affected patients. * Denotes a significant relation *p* < 0.05. *AFO*  ankle–foot orthosis

For the unilateral and bilateral affected subjects combined (n = 25, walking energy cost: r = − 0.264, *p* = 0.236, speed: r = 0.306, *p* = 0.132) and for bilateral affected subjects separately (n = 15, walking energy cost: r = 0.117, *p* = 0.679, speed: r = 0.07, *p* = 0.804), no significant relationships between difference in ankle power and difference in walking energy cost or speed were found. When only evaluating unilateral affected subjects, increase in ankle power with the experimental optimized AFO compared to the supplier recommended AFO stiffness related significantly with reduction in walking energy cost (n = 10, r = − 0.722, *p* = 0.018) (Fig. 3) and increase in speed (r = 0.709, *p* = 0.022).

## Discussion

In this study in persons with calf muscle weakness, we demonstrated that the supplier’s recommendation for the CA7 stiffness level results in the provision of DLS-AFOs that are stiffer and less effective compared to DLS-AFOs with a CA7 stiffness level that is individually optimized based on walking energy costs and 3D gait measurements. Although the recommended AFO stiffness improved energy cost while walking on level ground by 18.7% compared to no AFO, its reduction is only 82% of the effect that is achieved by experimentally optimizing the stiffness. Both ankle motion and ankle power were significantly larger with the experimental optimized AFO than the supplier recommended AFO.

The supplier recommended AFO stiffness based on body weight was only in 23% of the cases matched the experimentally selected optimal AFO stiffness. In most cases (n = 23 (67%)), the experimentally optimized AFO stiffness was more flexible, resulting in an additional absolute 5% reduction, in walking energy cost compared to the supplier recommended stiffness level. This effect is considered large and meaningful given that DLS-AFOs as provided in usual care reduce energy cost by 7 to 10% compared to no AFO, although the stiffness of dorsal leaf AFOs provided in usual care is often low [3, 17]. Moreover, although AFOs provided according to the supplier body weight-based algorithm reduced energy cost by 18% compared to no AFO, experimental optimization reduced energy cost by an additional 4.9% relative to the no AFO condition. This is a 22% improvement in effect of the AFO on energy cost compared to the body weight-based algorithm. This additional reduction is comparable with taking off a backpack of several kilograms [18, 19]. Contrary, walking speed increased only by a marginal and clinically unimportant 0.02 m/s (2%) compared to the supplier’s recommended AFO stiffness. This is in agreement with previous studies showing that speed is less sensitive to differences in stiffness than energy cost [5, 6].

The experimentally optimized AFO was more flexible and consequently resulted in a larger ankle range of motion and ankle power, which corresponds with previous work [5, 6]. Apparently, the negative consequences of a faster movement towards ankle dorsiflexion in late stance on energy cost were outweighed by its positive effects on increasing ankle power, resulting in a lower walking energy cost compared to the supplier recommended AFO. Increases in ankle power can reduce energy cost by lowering rebound work and necessary knee and hip compensations [1, 20, 21]. However, in our study the higher ankle power did not reduce compensatory hip power, despite lowering energy cost. In unilateral affected patients, increased ankle power related with energy cost reduction and increased speed, which may be more favored by patients than reductions in hip compensations. However, in bilateral affected subjects, such relation was absent and improvements in energy cost and speed are potentially likely due to positive effects on other factors less directly affected by AFO stiffness, such as trunk rotations, stability and knee flexion angles and moments during stance [22]. It needs to be assessed if indeed these measures are affected to a larger extent in bilateral affected patients in order to better understand how AFO stiffness affects energy cost in these subjects.

The aforementioned effects of experimental stiffness optimization clearly demonstrate that the selection of the individual optimal DLS-AFO stiffness solely on the basis of body weight is not sufficient to select the stiffness resulting in the best walking performance on level ground in people with calf muscle weakness. Other orthotic supplier’s recommend certain AFOs solely by type of weakness or impairment, but lack details on for example severity of weakness. Hence, such metrics are also unspecific and are unlikely to perform better in recommending the individual optimal stiffness. In order to improve the AFO stiffness provision, research should focus on creating comprehensive recommendations including all relevant factors to select the individual optimal AFO stiffness. Besides body weight and type of impairment, previous work indicated that higher walking speeds [10] and severity of (calf muscle) weakness [4, 23] influence gait biomechanics and the optimal stiffness. To determine the precise influence of these factors and their interactions on the optimal stiffness, simulations should be used as these, unlike human experiments, allow for independent and systematic manipulations of multiple subject-characteristics.

Meanwhile, to maximize walking performance on level ground, experimental stiffness optimization of CA7 dorsal leaf spring AFOs should be performed in clinical practice instead of following the suppliers’ recommendations, as long as they are not proven to be sufficient to predict the optimal stiffness. To reduce optimization time, costs and patient burden of extensive repetitive measurement procedures, we propose to limit the testing in usual care to the three stiffness levels between 2.8 and 4.3 Nm/degree (R5 to R3 of the CA7 series). These stiffness levels were optimal in almost 90% of our subjects, and higher stiffness levels were only optimal for subjects with a body weight above 80 kg and a slow walking speed without AFO of less than 1 m/s. Therefore, in these heavier and slower walkers, we advise the testing of stiffness levels 3.5 and 5.3 Nm/degree (R4 to R2 of the CA7 series). In the future, fast experimental optimizations might be achieved by human-in-the-loop optimizations [24], although such methods have currently not been used in clinical populations.

Although the current study demonstrates a large additional effect of optimizing the AFO stiffness on energy cost, part of this might be explained by the fact that we, among other variables, optimized towards energy cost. Additionally, we did not test whether the experimentally optimized AFO performed better in other walking conditions encountered during daily life. However, the experimentally optimized AFO was more flexible compared to the supplier recommended AFO and, therefore, imposes fewer restrictions in ankle motion. This likely leads to a better walking performance during locomotion conditions such as walking uphill and walking stairs, but meanwhile for a worse performance during conditions where the demands of the AFO are higher than during level walking, such as loaded walking. In short, the current study demonstrates that in people walking on level ground better outcomes can be achieved with AFOs more flexible than recommended by the orthotic supplier, but when prescribing an AFO stiffness in clinical practice individual circumstances and walking conditions should be taken into account.

## Conclusions

In people with calf muscle weakness, experimentally optimizing the CA7 stiffness outperforms current body weight-based supplier recommendations with regard to improvement in walking energy cost and speed. Current supplier recommendations result in the provision of AFOs that are stiffer than necessary and only achieve 80% of the potential reduction in energy cost, partly explained by the reduced ankle power associated with the higher stiffness. To better match the CA7 stiffness to the individual patient, thereby improving treatment outcomes, we recommend to experimentally optimize the AFO stiffness in people with calf muscle weakness. Additionally, our results suggest that precision orthotics, i.e. matching the AFO mechanics to the patient's pathomechanics, could potentially serve a wider range of patients.

## Supplementary Information

**Additional file 1: Table.** Individual effects of the experimentally optimized stiffness versus the supplier recommended stiffness.

## Data Availability

The datasets used and/or analyzed during the current study are available from the corresponding author on reasonable request.
