# Supplementary material for: Individual stiffness optimization of dorsal leaf spring ankle–foot orthoses in people with calf muscle weakness is superior to standard bodyweight-based recommendations
Source: J Neuroeng Rehabil. 2021 Jun 8;18:97. doi: 10.1186/s12984-021-00890-8 (PMC8186056; doi:10.1186/s12984-021-00890-8)
Supplement: Supplementary file 1 — Additional file 1: Table. Individual effects of the experimentally optimized stiffness versus the supplier recommended stiffness. [file 12984_2021_890_MOESM1_ESM.pdf]

Supplementary table. Individual effects of the experimentally optimized stiffness versus the supplier recommended stiffness.

Subject	Unilateral / bilateral affected	Supplier recommend stiffness	Experimentally optimized stiffness	difference EC (J/kg/m)	difference speed (m/s)	difference ankle power (W/kg)
P01	Unilateral	6.6	5.3	-.25	.02	-.16
P02	Bilateral	3.5	6.6	-.17	-.01	.41
P03	Unilateral	2.8	5.3	.05	.00	-.05
P04	Unilateral	4.3	5.3	-.29	.02	.26
P05	Bilateral	4.3	6.6	-.30	.04	.59
P06	Bilateral	4.3	5.3	-.27	.11	-.03
P07	Bilateral	3.5	6.6	.07	-.01	.09
P08	Bilateral	4.3	5.3	-.11	.06	.05
P09	Unilateral	3.5	3.5	.00	.00	.00
P10	Bilateral	6.6	6.6	.00	.00	.00
P11	Unilateral	3.5	6.6	-.46	.12	.56
P12	Bilateral	5.3	5.3	.00	.00	.00
P13	Bilateral	3.5	3.5	.00	.00	.00
P14	Bilateral	3.5	4.3	-.40	.00	.22
P15	Unilateral	3.5	5.3	-.72	.12	.28
P16	Unilateral	6.6	6.6	.00	.00	.00
P17	Unilateral	3.5	6.6	-.79	.08	.45
P18	Unilateral	2.8	3.5	-.11	.03	.10
P19	Bilateral	3.5	5.3	-.11	.05	.36
P20	Bilateral	2.8	6.6	-.35	-.04	-.01
P21	Bilateral	4.3	6.6	-.30	.10	.28
P22	Bilateral	2.8	4.3	-.45	.04	.31
P23	Unilateral	3.5	2.8	.08	.04	-.23
P24	Unilateral	2.8	5.3	-.47	.04	-.02
P25	Bilateral	4.3	4.3	.00	.00	.00
P26	Bilateral	4.3	5.3	-.41	.02	.63
P27	Bilateral	2.8	4.3	-.92	-.02	.01
P28	Bilateral	4.3	2.8	-.20	.01	Not measured
P29	Bilateral	3.5	3.5	.00	.00	Not measured
P30	Unilateral	4.3	3.5	-.13	-.04	-.06
P31	Bilateral	5.3	6.6	-.27	.08	.33
P32	Bilateral	4.3	4.3	.00	.00	.00
P33	Bilateral	2.8	4.3	.04	-.01	.20
P34	Bilateral	3.5	6.6	.14	-.02	.37
